# Physical Activity, Diabetes, and Heart Conditions Among Native Hawaiian and Other Pacific Islander Older Adults

**DOI:** 10.1093/geroni/igaa057.1298

**Published:** 2020-12-16

**Authors:** Yeonjung (Jane) Lee, Tyran Terada

**Affiliations:** University of Hawaiʻi at Mānoa, Honolulu, Hawaii, United States

## Abstract

The Native Hawaiian and Other Pacific Islander (NHOPI) older adult population remains understudied and are disproportionately affected by diabetes and heart conditions. Research has shown that participating in physical activity is a protective factor for many of the health conditions experienced by older adults. Yet, the link between physical activity, diabetes, and heart conditions among the NHOPI older adult population is limited. The purpose of this study is to identify the characteristics of NHOPI older adults and to explore the association between physical activity levels and diabetes/heart conditions. Methods and findings Using data from the 2014 Native Hawaiian and Pacific Islander National Health Interview Survey (NHPI NHIS), which is considered to have a representative sample of NHOPI, the study explores the associations between physical activity and diabetes/heart conditions. A total of 1,045 older adults ages 50 years and older were included for analyses. Weighted multivariate analyses with multiple imputation techniques were used. The NHPI NHIS is the first federal survey focusing on the NHOPI population of the United States with rich information on health. Results and Implications Those with who were engaged in a sufficient physical activity had lower odds of having diabetes or heart conditions than their counterparts without physical activity while controlling for other sociodemographic characteristics. Findings highlight the importance of physical activity promotion intervention in preventing cardiovascular disease. Research and practice addressing health disparities and cardiovascular conditions should leverage efforts to provide culturally relevant physical activity types and resources to NHOPI older adults.

